# In-depth proteomic profiling captures subtype-specific features of craniopharyngiomas

**DOI:** 10.1038/s41598-021-00483-4

**Published:** 2021-10-27

**Authors:** Jung Hee Kim, Hyeyoon Kim, Kisoon Dan, Seong-Ik Kim, Sung-Hye Park, Dohyun Han, Yong Hwy Kim

**Affiliations:** 1 Department of Internal Medicine, Seoul National University Hospital, Seoul, Republic of Korea; 2 Pituitary Center, Seoul National University Hospital, Seoul, Republic of Korea; 3 Proteomics Core Facility, Biomedical Research Institute, Seoul National University Hospital, 101 Daehak-ro, Jongno-gu, Seoul, 03080 Republic of Korea; 4 Department of Pathology, Seoul National University Hospital, Seoul, Republic of Korea; 5 Transdisciplinary Department of Medicine & Advanced Technology, Seoul National University Hospital, Seoul, Republic of Korea; 6 Department of Neurosurgery, Seoul National University Hospital, 101 Daehak-ro, Jongno-gu, Seoul, 03080 Republic of Korea

**Keywords:** Cancer genetics, CNS cancer, Surgical oncology

## Abstract

Craniopharyngiomas are rare epithelial tumors derived from pituitary gland embryonic tissue. This epithelial tumor can be categorized as an adamantinomatous craniopharyngioma (ACP) or papillary craniopharyngioma (PCP) subtype with histopathological and genetic differences. Genomic and transcriptomic profiles of craniopharyngiomas have been investigated; however, the proteomic profile has yet to be elucidated and added to these profiles. Recent improvements in high-throughput quantitative proteomic approaches have introduced new opportunities for a better understanding of these diseases and the efficient discovery of biomarkers. We aimed to confirm subtype-associated proteomic changes between ACP and PCP specimens. We performed a system-level proteomic study using an integrated approach that combines mass spectrometry-based quantitative proteomic, statistical, and bioinformatics analyses. The bioinformatics analysis showed that differentially expressed proteins between ACP and PCP were significantly involved in mitochondrial organization, fatty acid metabolic processes, exocytosis, the inflammatory response, the cell cycle, RNA splicing, cell migration, and neuron development. Furthermore, using network analysis, we identified hub proteins that were positively correlated with ACP and PCP phenotypes. Our findings improve our understanding of the pathogenesis of craniopharyngiomas and provide novel insights that may ultimately translate to the development of craniopharyngioma subtype-specific therapeutics.

## Introduction

Craniopharyngiomas (CPs) are benign brain tumors (WHO I) constituting of 2–5% of all brain tumors. However, they are among the most challenging intracranial tumors to treat due to their tendency frequently recur despite gross total removal and the critical neurovascular structures surrounding the location of the tumor origin^1^. Two histopathological phenotypes, adamantinomatous craniopharyngioma (ACP) and papillary craniopharyngioma (PCP), are distinctive clinical entities with dissimilar theories of tumorigenesis^2,3^. ACP is found in people of all ages, and the embryogenic theory explains that the pathogenesis is the result of the neoplastic transformation of the embryonic squamous epithelial nest in the craniopharyngeal duct, which bridges Rathke’s pouch to the stomodeum, forming tooth primordia. In the developmental process in which the Rathke’s pouch forms the adenohypophysis, cell remnants of the craniopharyngeal duct migrate to the sellar and suprasellar regions, the most frequent location of CPs. The histopathological feature of ACP is a palisading squamous epithelium with nodules of wet keratin resembling enamel-forming neoplasms in the oropharynx, and the calcification observed in brain images is a common feature that is found in approximately 90% of ACP patients. In contrast, PCP, which exclusively affects adults, is recognized by the monomorphous mass of well-differentiated squamous epithelium with papillary projection observed in a microscopic examination and is thought to be generated by the metaplasia of squamous epithelial cells that are remnants of the part of the stomodeum that contributed to the buccal mucosa, which is the metaplasia theory.

Recent advancements have revealed the different molecular signatures of the two CP types. Mutations in the CTNNB1 gene, encoding β-catenin, are exclusively found in human ACPs, but not in the papillary subtype, and a mouse model with a degradation-resistant β-catenin mutant developed a tumor in the pituitary gland upon overactivation of the Wnt pathway in ACP^4,5^. On the other hand, V600E point mutations in BRAF, encoding B-Raf, a cytosolic kinase in the mitogen-activated protein kinase (MAPK) pathway, were recently discovered in approximately 95% of PCPs^6–8^. Targeted therapy with B-Raf inhibitors showed a dramatic response in patients with PCP, similar to that seen in melanomas; however, the duration of the therapy needed to achieve complete remission remains to be determined^9,10^. Moreover, a few genomics studies based on gene expression analysis have provided comprehensive insight into the molecular pathogenesis and distinguishing features of both CP subtypes^11,12^. However, a systems-wide understanding of the distinct molecular features of ACP and PCP using mass spectrometry (MS)-based proteomics is limited.

MS-based quantitative proteomics is a powerful tool that can offer unprecedented insights into disease-related molecular and cellular processes^13^. Recently, two strategies, label-free quantification (LFQ) and isobaric labeling with tandem mass tag (TMT), have become popular for protein-wide quantification^14^. In this study, we utilized LFQ proteomics complemented with a TMT isobaric labeling proteomic approach to identify the global proteomic changes in ACP and PCP. Furthermore, we identified biological processes according to subtype-related protein expression patterns to gain new insight into the pathogenesis of CP.

## Results

### Clinical characteristics of the study subjects

Six subjects with ACP (mean age, 50.0 years; female, n = 3; male, n = 3) and six subjects with PCP (mean age, 48.1 years; female, n = 3; male, n = 3) were enrolled in the present study (Table 1). The tumor volumes were larger in the subjects with ACP than in those with PCP. However, there was no difference in clinical features between the two subtypes. One papillary tumor sample was discarded during protein extraction process due to blood contamination (Supplementary Table S1 online).

**Table 1:** Baseline characteristics of the study subjects.

Variables	ACP (n = 6)	PCP (n = 6)	*P* value
Age (years)	50.0 ± 18.3	49.3 ± 18.1	0.954
Gender (female, %)	3 (50.0%)	3 (60.0%)	0.740
Maximal tumor size (cm)	2.42 ± 0.81	3.22 ± 0.66	0.111
Tumor volume (cm^3^)	3.91 ± 3.48	10.6 ± 5.74	0.041
Third ventricle invasion	4 (66.7%)	4 (80.0%)	0.621

Data are shown as the mean ± standard deviation or n (%).

ACP, adamantinomatous craniopharyngioma; PCP, papillary craniopharyngioma.

### Overall scheme of the proteome profiling analysis of the craniopharyngioma subtypes

**Figure 1:**
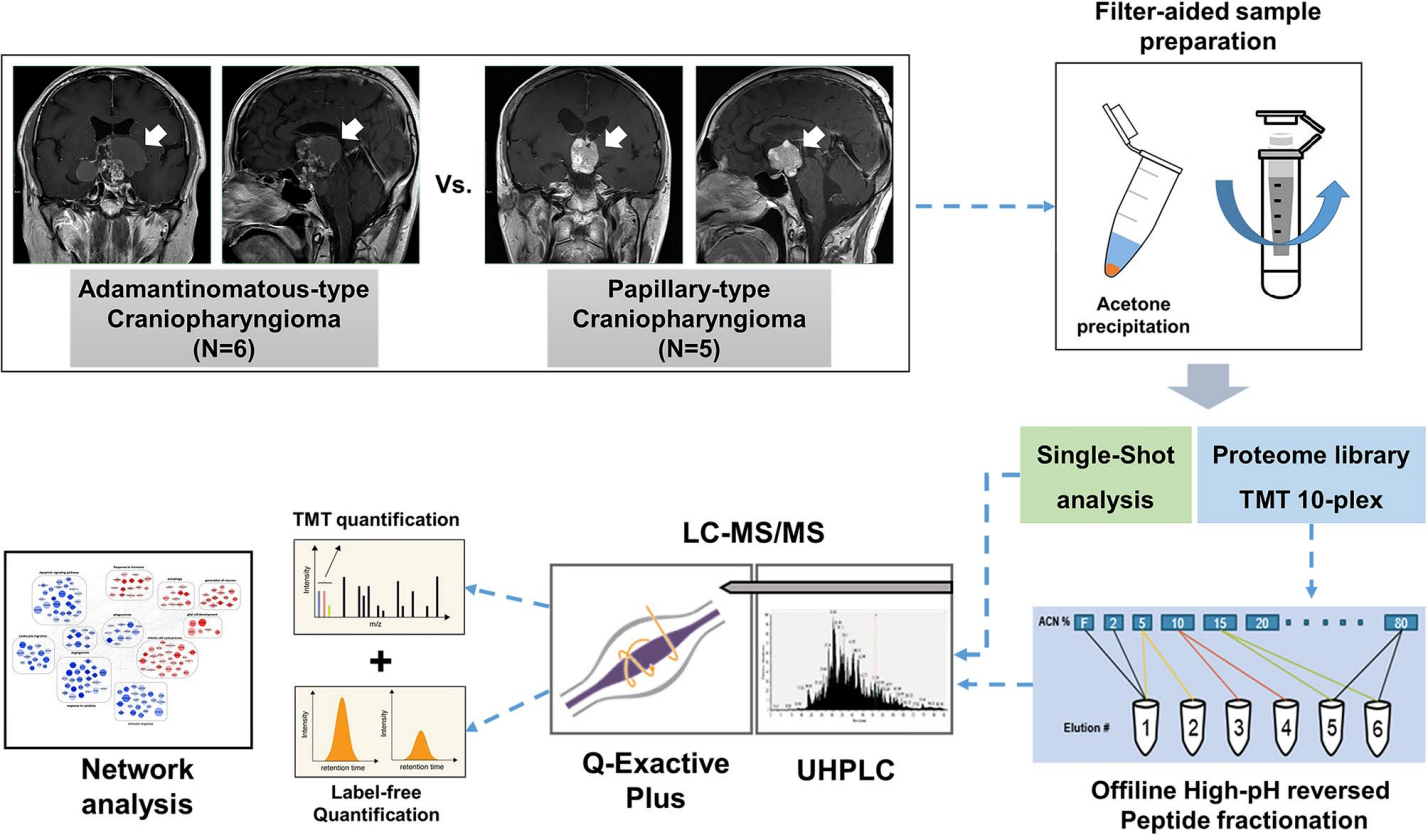
Overall scheme of the quantitative proteomic analysis of craniopharyngioma. Schematic showing the proteomic analysis of craniopharyngiomas (ACPs and PCPs) analyzed by label-free quantification and TMT labeling experiments.

To identify proteome signatures in the ACP and PCP samples, we performed two quantitative proteomic analyses, including the label-free quantification method (LFQ) and the tandem mass tagging (TMT) method (Fig. 1). Briefly, tissue lysates were digested via filter-aided sample preparation (FASP), which yielded 40–110 μg of peptides from frozen tissues (Supplementary Table S1 online). First, label-free quantification was performed using unfractionated peptide digests (20 μg per samples) from freshly frozen tumors (6 ACPs and 5 PCPs). It included a single-shot LC–MS/MS analysis of digested peptides using long gradients to identify peptides with high quantitative accuracy. Except for one ACP and one PCP sample due to limited sample amount, TMT 10-plex quantification was performed using the same samples that had been analyzed via label-free quantification (5 ACPs and 4 PCPs). After each peptide sample (40 μg per samples) was labeled with TMT 10-plex reagent, the labeled peptide mixtures were separated into 12 fractions using high-pH reversed-phase fractionation and analyzed in 3-h LC–MS/MS runs on the Q-Exactive Plus system.

### Label-free quantification

**Figure 2:**
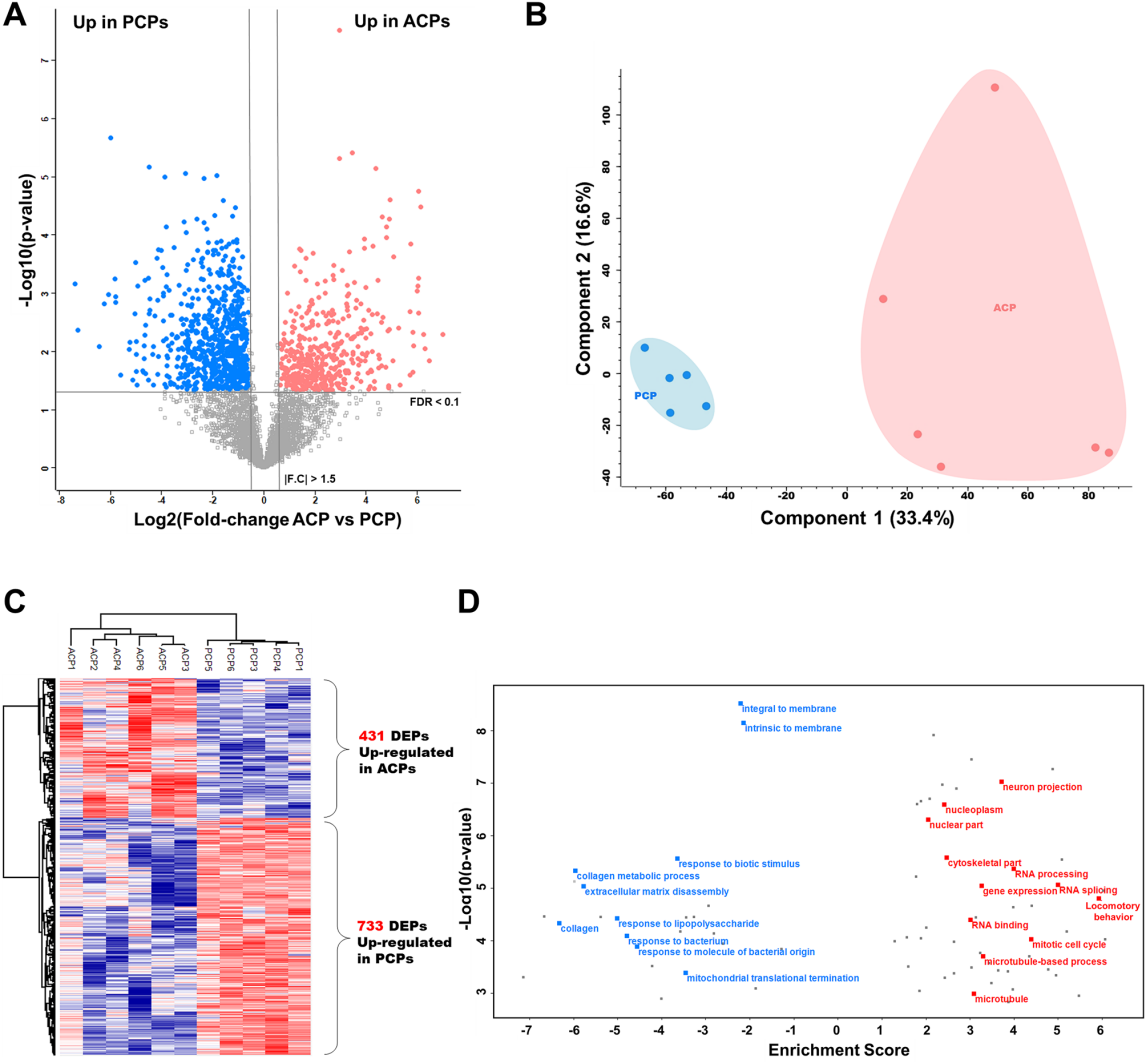
Results of the label-free quantification approach. (**A**) Volcano plot depicts differential expression between two craniopharyngioma subtypes (ACP and PCP) using label-free quantification data. (**B**) Principal component analysis of the 11-tumor label-free proteomic data. (**C**) The heatmap represents unsupervised hierarchical clustering of the 11 tumor samples using 923 proteins that were differentially expressed as identified by label-free quantification. (**D**) Gene ontology (GO) enrichment analysis of the label-free data. The red points indicate upregulated proteins in ACPs, and blue points indicate upregulated proteins in PCPs.

In total, the LFQ method resulted in the identification of 49,146 unique peptides corresponding to 5464 protein groups with a protein FDR level < 1% (Supplementary Table S2 online). For our data, we considered only a subset of 4204 proteins with quantitative valid values across at least 70% of the samples. For a functional view of the proteomic data, we used volcano plots to compare expression differences between the ACP and PCP samples. Considering t-test results for pairwise comparisons and employing a filtering strategy based on a permutation-based FDR < 0.1 and fold-change > 1.5, we identified 1164 proteins that showed significant differential expression, of which 431 were upregulated in the ACP samples and 733 were upregulated in the PCP samples (Fig, 2A,B, and Supplementary Table S3 online). To assess the quantitative reproducibility between biological replicates, we calculated the average Pearson correlation coefficients within and between groups. Interestingly, the biological correlation data indicated higher diversity among the ACP samples (average Pearson correlation = 0.76) compared with the PCP samples (average Pearson correlation = 0.88) (Supplementary Fig. 1 online). Hierarchical clustering and principal component analysis (PCA) revealed tight clustering of the ACP and PCP samples and their corresponding biological replicates, indicating distinct protein expression patterns within each subtype (Fig. 2B,C). To analyze the functional differences between the ACP and PCP samples, we performed 1D annotation enrichment analysis based on GO using Perseus software^15^, and the results revealed 81 statistically significant features with a Benjamini–Hochberg FDR < 0.05 (Supplementary Table S4 online). The terms related to locomotory behavior, RNA processing, gene expression, the mitotic cell cycle, neuron projection, and microtubule-related process were mainly enriched in ACP subtype, while the terms associated with membrane, extracellular matrix disassembly, response to lipopolysaccharide or bacterium, and mitochondrial activity were significantly enriched in the PCP subtype (Fig. 2D).

### Quantitative analysis based on TMT 10-plex

**Figure 3:**
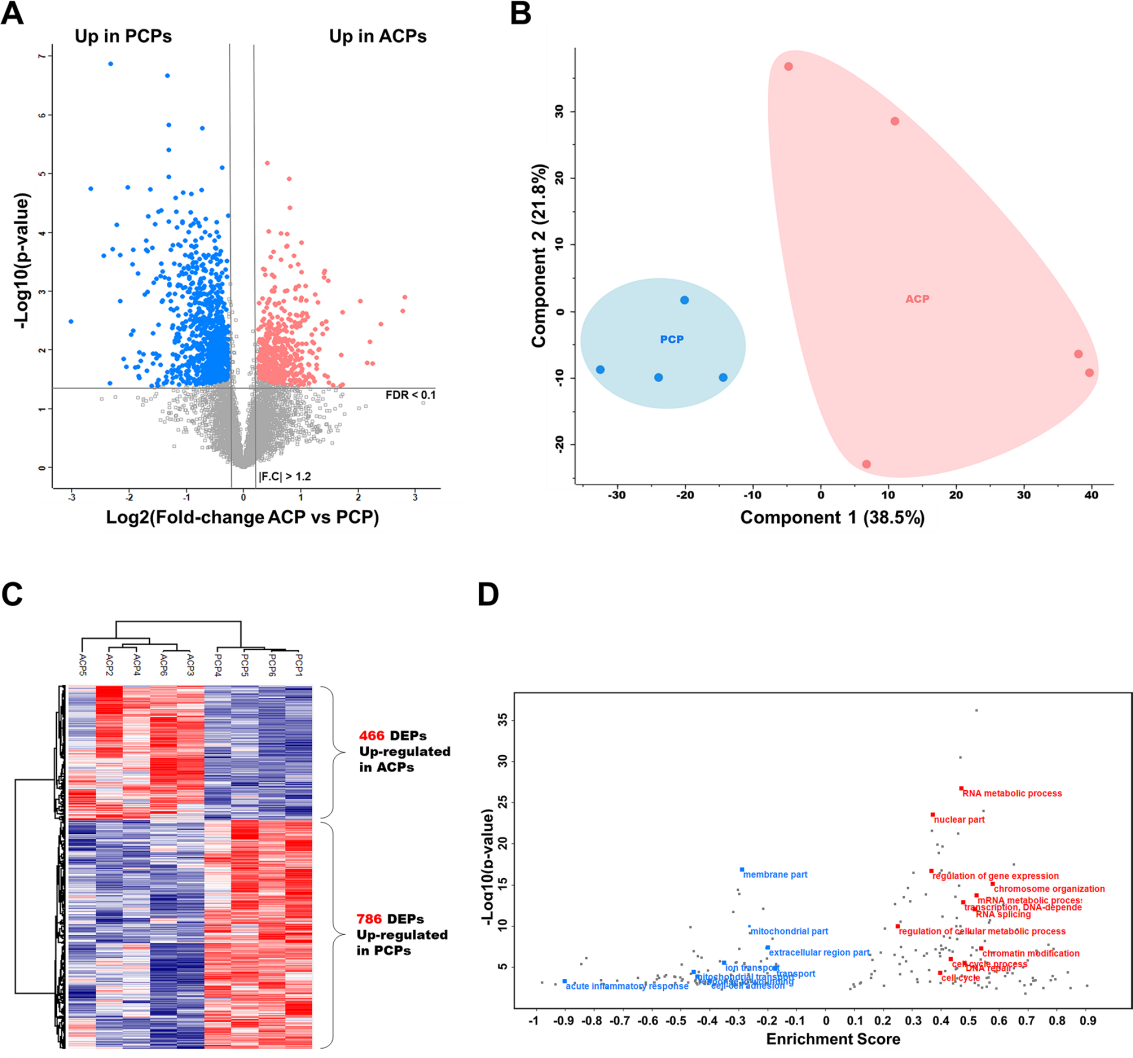
Results of—TMT quantification approach. (**A**) Volcano plot depicting the differential expression analysis of the two craniopharyngioma subtypes (ACP and PCP) using TMT quantification data. (**B**) Principal component analysis of the 9-tumor TMT quantification data. (**C**) The heat map represents unsupervised hierarchical clustering of the 9 tumor samples using 1474 proteins that were differentially expressed and identified in the TMT quantification data. (**D**) Gene ontology (GO) enrichment analysis of TMT quantification data. The red points indicate upregulated proteins in ACPs, and blue points indicate upregulated proteins in PCPs.

The MS analysis of the TMT 10-plex experiment yielded 6874 identified protein groups and 6664 protein groups quantified with high confidence at the 1% level based on the protein FDR (Supplementary Table S5 online). We observed an excellent correlation between biological replicates (average R = 0.94–0.98) (Supplementary Fig. 2 online). Analysis of a spiked ovalbumin standard protein used for batch normalization revealed only a small variation (CV < 1%) during processing, indicating that the quantified expression diversity stems from true biological differences between subtypes. TMT quantification based on MS2 reporter ion intensity led to the identification of 1252 differentially expressed proteins with a permutation-based FDR < 0.1 and fold-change > 1.2 (Fig. 3A and Supplementary Table S6 online). Hierarchical clustering and PCA revealed tight clustering of the two subtypes and the corresponding biological replicates, indicating distinct protein expression patterns within each group (Fig. 3B,C). Consistently, samples from the PCP patients were closely associated in the PCA compared with those from the ACP patients. The 1D annotation enrichment showed that the upregulated proteins in ACP were functionally enriched in RNA metabolic process, regulation of gene expression, and the cell cycle (Fig. 3D and Supplementary Table S7 online). In PCP, proteins were mainly enriched in membrane proteins, extracellular regions, mitochondrial activity, responses to wounding and acute inflammatory responses, which is consistent with the results of the enrichment analysis using DEPs obtained from the label-free quantification.

### Functional enrichment analysis was performed by combining two quantification strategies

**Figure 4:**
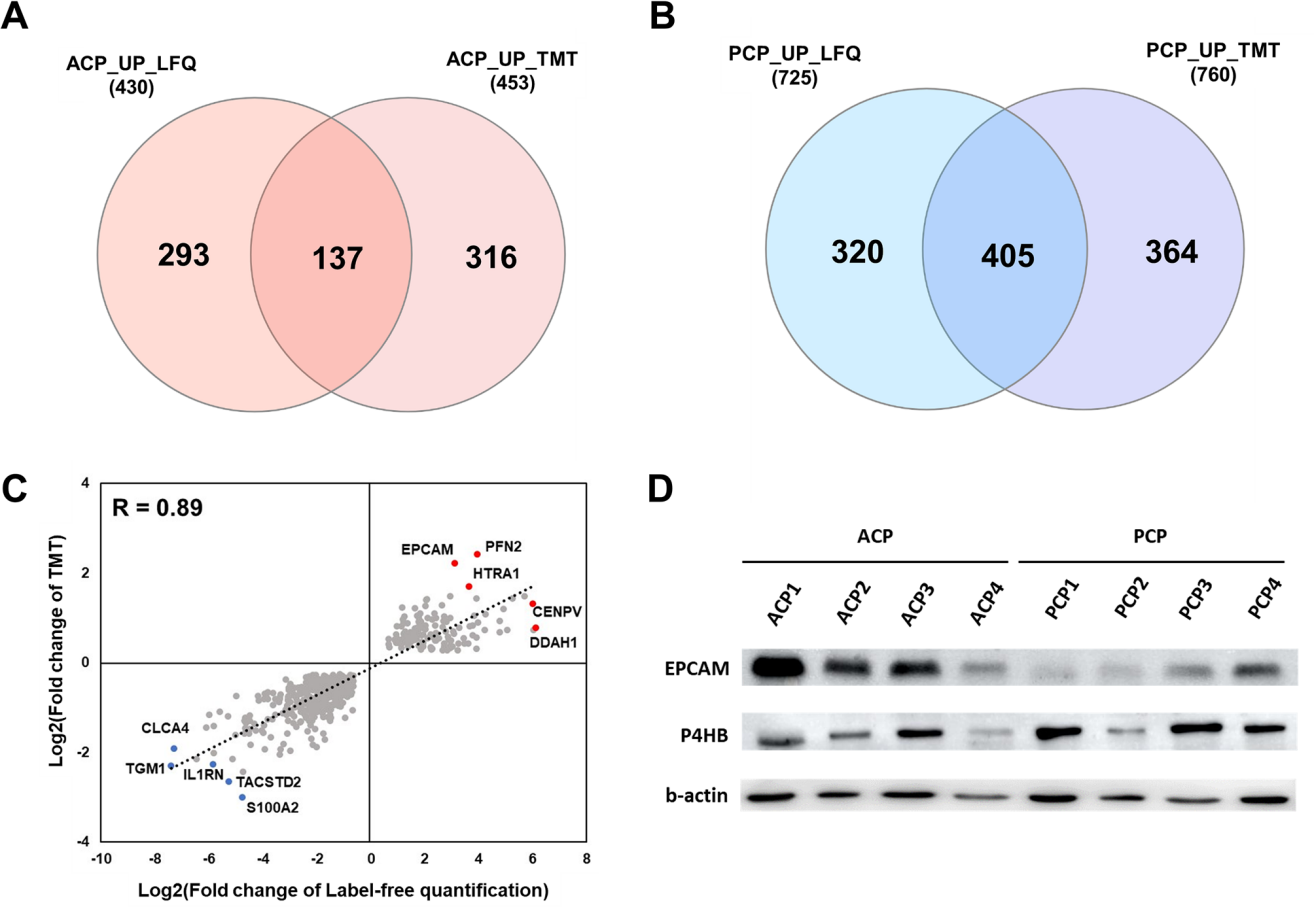
Comparison between label-free and TMT quantification methods. (**A**) Venn diagram showing the comparison of differentially upregulated proteins between ACPs in both datasets. (**B**) Venn diagram showing the comparison of differentially upregulated proteins between PCPs in both datasets. (**C**) Correlation analysis of fold changes in the label-free and TMT quantification data. (**D**) Validation of the proteomic data using protein expression. Western blotting was performed to measure EPCAM, P4HB and β-actin.

To select DEPs with consistent results based on these two different quantitative methods, we analyzed the overlap of the DEP sets in each subtype. The overlap between LFQ and TMT in the ACP group was relatively small (18.4% of all DEPs in the ACP group). In contrast, DEP overlap in the LFQ and TMT data of the PCP group was 37.2% (405 proteins). We found a strong correlation (R = 0.89) between fold-changes of DEPs identified by both LFQ and TMT quantification strategies, suggesting that our quantitative analysis is highly reproducible and reliable (Fig. 4). To validate the results of our quantitative proteomic analysis, we performed Western blot analysis with a set of ACP samples (n = 4) and PCP samples (n = 4). First, EPCAM was selected for validation as the previously identified markers of adamantinomatous craniopharyngioma^16,17^. Additionally, one protein (P4HB) was randomly selected from the up-regulated proteins in papillary craniopharyngioma. The expression levels of EPCAM and P4HB were increased in the ACP and PCP sample sets, respectively (Fig. 4D and Supplementary Fig. 3 online), indicating that Western blot substantially verified the expressional differences first obtained by MS.

**Figure 5:**
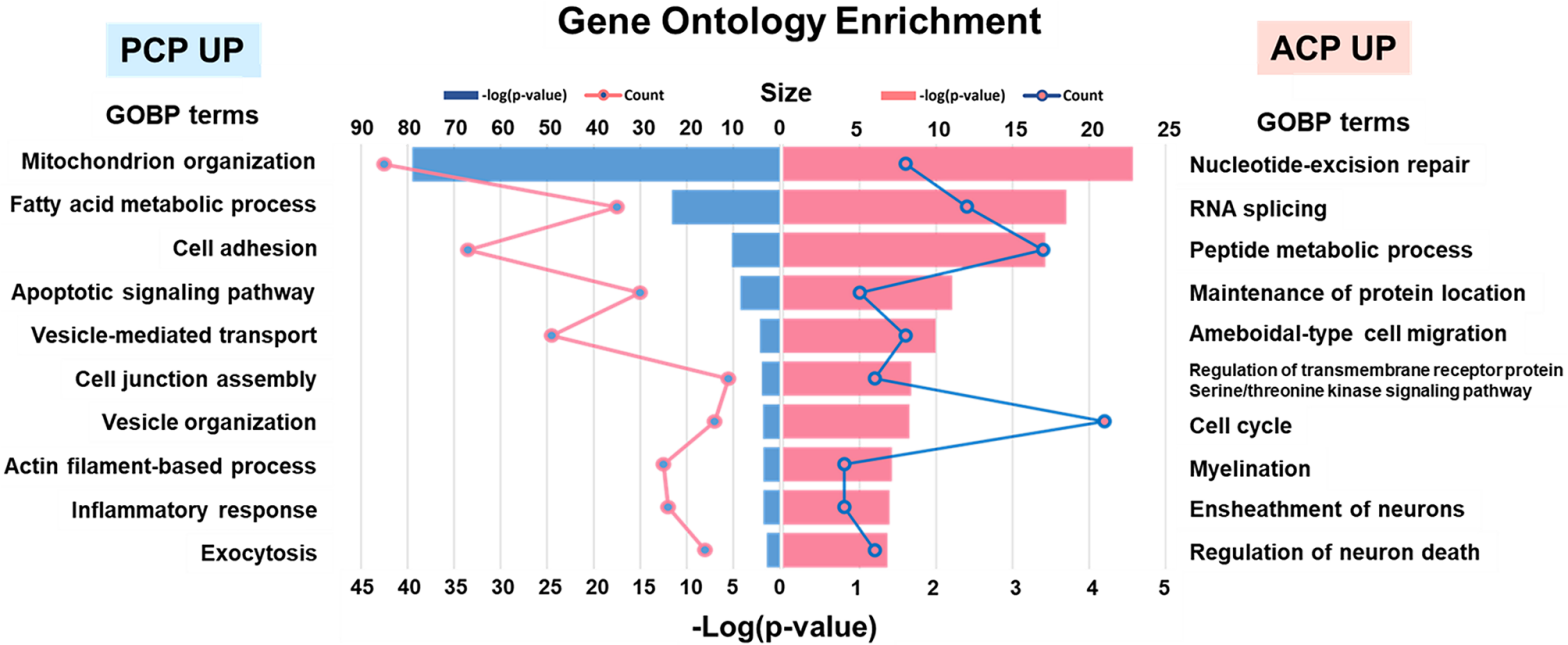
Comparison analysis of gene ontology enrichment (biological process category) between ACP and PCP. GO analysis of the common differentially expressed proteins. The bar graph represents the enrichment score, − log_10_ (p-value), as heights. Each line illustrates the number of proteins enriched per GO term.

Enrichment analysis based on GO using the overlapping DEPs (total 542 proteins) was performed to identify the common cellular processed and pathways between the proteins identified in the TMT and label-free quantification experiments. Fischer’s exact test was used to identify numerous biological processes enriched among the DEPs (p < 0.05; Fig. 5 and Supplementary Table S8 online). This analysis revealed that DEPs representing distinct biological processes were significantly enriched in the different CP subtypes. Proteins that were upregulated in ACP were associated with nucleotide-excision repair, RNA splicing, the cell cycle, cell migration, and nervous system development. In the case of the upregulated proteins in PCP, mitochondrial organization, fatty acid metabolic process, cell adhesion, apoptotic signaling pathway, vesicle organization, inflammatory response, and exocytosis were significantly enriched.

**Figure 6:**
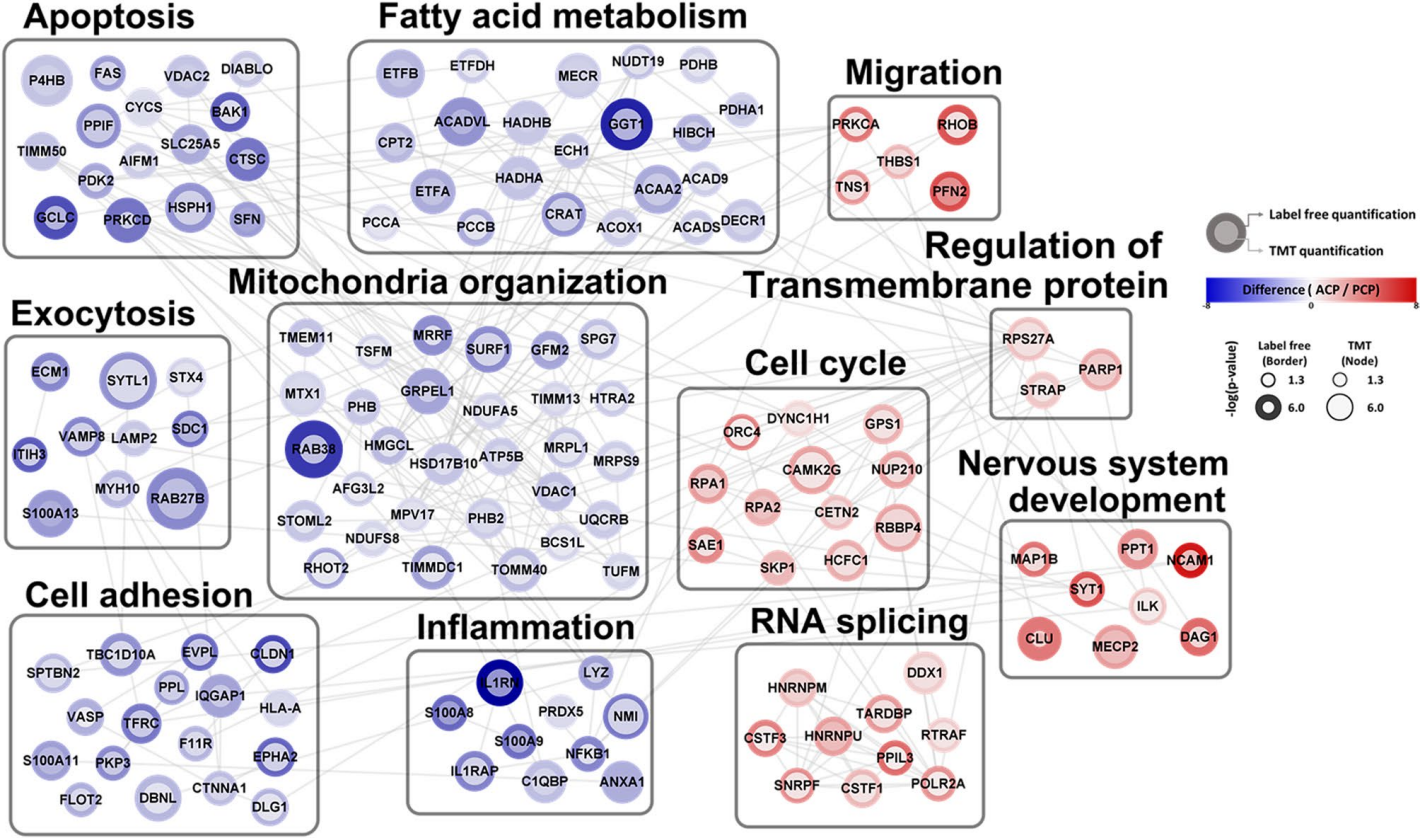
Network model of proteome characterization between ACP and PCP. A protein interaction network model was generated by integrating two proteomic datasets. Red nodes represent upregulated expression in ACPs, and blue nodes represent upregulated expression in PCPs. The color of the outer node indicates the differential expression levels revealed through the label-free quantification, and the color of the inner node indicates the differential expression level revealed through the TMT quantification. The gray line represents protein–protein interactions derived from the STRING database.

To explore the collective functions of the DEPs according to subtype, we constructed a network model to describe the interactions among the DEPs involved in the GO BPs specifically enriched in each group (Fig. 6). First, the network model showed the upregulation of several processes that are known to be associated with the nucleus, including the cell cycle (DYNC1H1, GPS1, TUBA1A, RBBP4, BCAT1, SAE1, SKP1, RPA1, RPA2, HCFC1, NUP210, and CAMK2G) and RNA splicing (CSTF1, CSTF3, SNRPF, HNRNPU, HNRNPM, DDX, TARDBP, POLR2A, RTRAF, and PPIL3). The network model further showed that the nervous system development (MAP1B, MECP2, PPT1, NCAM1, SYT1, CLU, DAG1, and ILK) were upregulated in ACP. Finally, the network model revealed upregulation of cell migration (PRKCA, TNS1, THBS1, RHOB, and PFN2) and regulation of transmembrane proteins (PARP1, STRAP, and RPS27A) in ACP compared with PCP.

In contrast, the network model shows an upregulation of pathways related to mitochondrial functions, including mitochondrial organization and fatty acid metabolism, in PCP compared with ACP (Fig. 6). Consistent with the upregulation of these pathways, the network model further showed that exocytosis (RAB27B, ECM1, STYL1, SDC1, ITIH3, S100A13, MYH10, LAMP2, VAMP8, and STX4) and inflammation (S100A8, S100A9, NMI, C1QBP, IL1RAP, IL1RN, NFKB1, ANXA1, PRDX5, and LYZ) were also upregulated in PCP. Additionally, the proteins associated with apoptosis were largely upregulated in PCP, suggesting complex alteration patterns in the mitochondrial pathway of apoptosis^18^. Moreover, the network model showed the upregulation of many proteins involved in cell adhesion. Collectively, the upregulation of these key mitochondrial-associated processes in the network model suggests changed mitochondrial function and activity in PCP compared with ACP.

**Figure 7:**
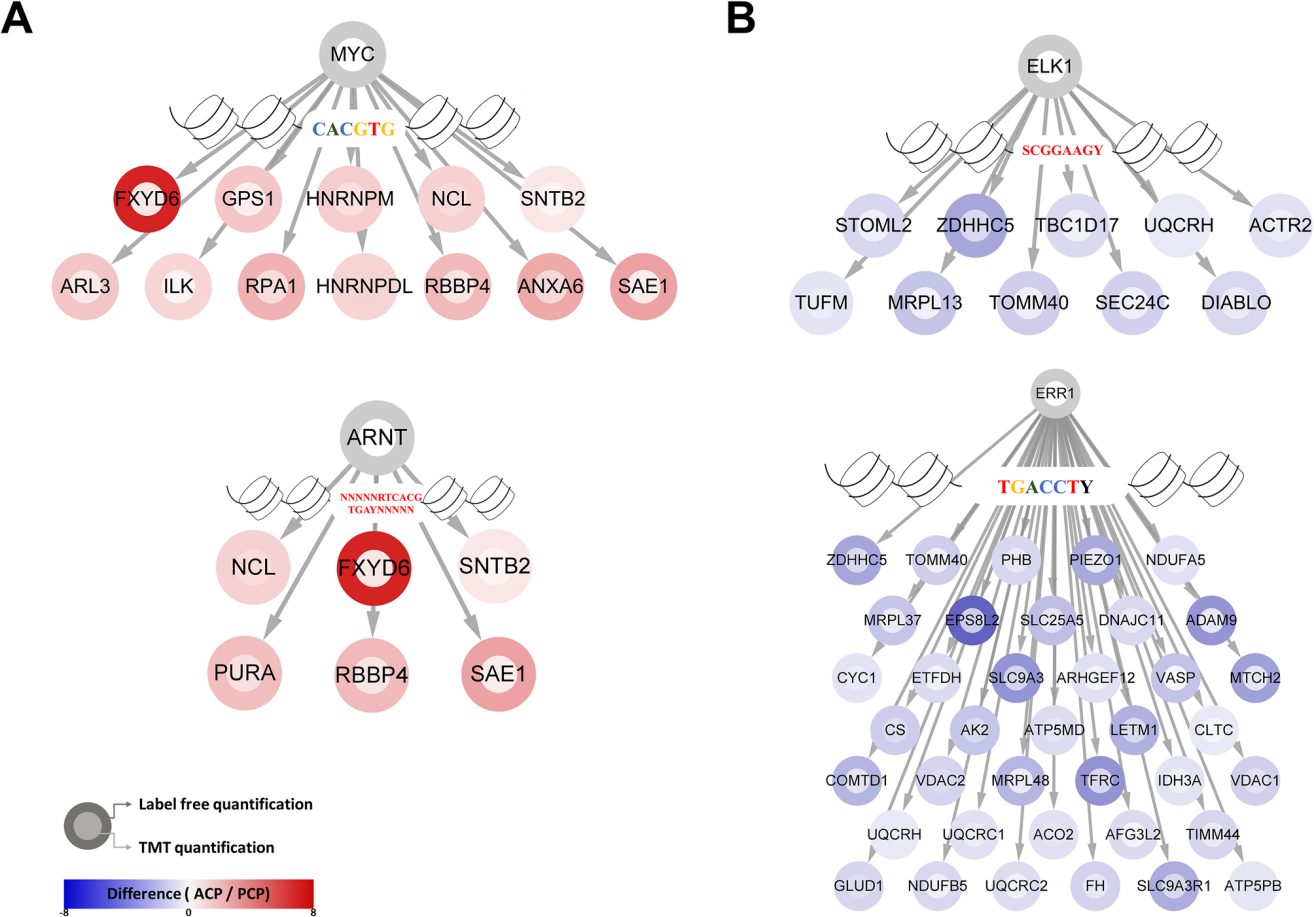
Prediction of the binding sites of transcription factors and the network of TFs and downstream targets. (**A**) Predicted TFs (MYC and ARNT) in ACPs. (**B**) Predicted TFs (ELK1 and ERR1) in PCPs. The color of the outer node indicates the differential expression levels revealed through label-free quantification, and the color of the inner node indicates the differential expression level revealed through TMT quantification.

To identify transcription factors (TFs) that can drive the subtype-related changes in protein expression observed in our proteomic analysis, we predicted transcription factor binding sites (TFBSs) in DEPs and selected candidate regulators. The upregulated proteins in ACP showed TF BS enrichment with USF1, MYC, E2F1, ARNT, and NRF1 in both quantitative proteomic datasets (Supplementary Table S9 and Supplementary Fig. 4 online). For example, several MYC target proteins (ANXA6, ARL3, SNTB2, ILK, HNRNPM, FXYD6, NCL, HNRNPDL, RBBP4, GPS1, SAE1, and RPA1) were significantly enriched in ACP (Fig. 7A). In addition, NCL, FXYD6, SNTB2, SAE1, PURA, and RBBP4 were enriched in ACP in both datasets as ARNT downstream targets. The proteins in PCP that were upregulated compared with those in ACP showed TFBS enrichment, including SF1, ERR1, NRF1, NRF2, ER, and ELK1 (Supplementary Table 9 online). In the case of ELK1, TOMM40, STOML2, MRPL13, TUFM, UQCRH, DIABLO, ZDHHC5, SEC24C, ACTR2, and TBC1D17 were observed to be significantly enriched with ELK1 targets in both proteomic datasets (Fig. 7B). Moreover, ERR1 was predicted to have 38 downstream target proteins.

## Discussion

Our molecular understanding of CP has been largely dominated by omics-based profiling approaches such as genomics, transcriptomics, and proteomics. Given the functional role of proteins in cellular processes, complementary approaches that integrate global, unbiased, translational readouts with well-characterized genomic events will likely substantially improve our understanding of the pathogenetic mechanisms of CP. Two different subtypes of CPs must be distinguished according to the current WHO classification of CNS tumors. ACPs and PCPs differ in their pathognomonic features, age distribution, clinical course, and gene mutations^19^. Although genetic analysis has identified specific pathway alterations and gene mutations associated with CP pathogenesis, the underlying molecular mechanism(s) remains to be fully clarified, with the subsequent identification of effective targets of therapy. Additionally, discrimination of CP subtypes is often challenging when only small and/or fragmented surgical specimens are used, and CP with a mixed histological pattern has been specified and promoted in several reports^20^. Newly described molecular markers may help to resolve this problem, and recent omics-based approaches based on large numbers and well-characterized tumor samples are required to determine the important implications for a differential diagnosis and treatment of CP. In contrast to previous proteomic research, we compared the two subtypes of CPs instead of using Rathke’s cleft cysts or normal brains.

While accumulating studies are now beginning to reveal the CP proteome, our study is the first in-depth systems-side quantitative proteomic study of CP to date, and it shows how proteomics can add to our understanding of subtype-specific changes in protein expression and driving mechanisms. First, we performed two different quantitative proteomic analyses, providing important insights into subtype differences and thus extending our views obtained from previous genomics studies^6,11^. Next, we used this approach to propose biological pathways operational in distinct CP types. The GO enrichment and network analyses revealed entire protein networks that are unique to each CP subtype.

ACPs are driven by somatic mutations in *CTNNB1* (encoding β-catenin) that affect β-catenin stability and predominantly appear as cysts^4^. The CTNNB1 mutation leads to the activation of Wnt-regulated cellular processes^19,21^. First, the Wnt pathway is the key pathway in the activation of the cell cycle^22^ and cell migration^23^. Furthermore, many reports have emphasized that the EGFR and SHH signaling pathways are also upregulated in ACPs and are associated with tumor cell migration^24,25^. As expected, ACP proteins that upregulated compared to PCP proteins were highly enriched in the cell cycle, RNA splicing, neural development, and migration. In particular, EPCAM protein levels revealed clear differences in the results of both proteomic approaches. EPCAM, known as a target of Wnt/β-catenin, was previously reported to be differentially expressed in CP subtypes^16^. Our results further support the involvement of EPCAM in Wnt-activating tumors. In addition, we found markedly higher levels of several Wnt-related proteins (NCAM1, STRAP, and MECP2) in ACPs. We also demonstrated that the serine/threonine kinase signaling pathway was upregulated in ACPs.

Our proteomic profiling may characterize the histopathological structures of ACPs. ACPs consist of solid and cystic components. The solid component is the epithelial tumor, which is composed of pseudostratified palisading epithelium, stellate reticulum, and whorl-like structures. The palisading epithelium protrudes in a finger-like shape and invades into surrounding tissues^26^. The GO terms ameboidal-type cell migration and dendrite development enriched in ACPs may reflect the protrusion of the tumor epithelium. Our network analysis also revealed the pathway of neuron and glial cell development. This GO term may implicate glial reactive tissues, which surround epithelial tumors and consist of astrocytes and immune cells^27^. However, we did not include proteins from the cystic component, which included several inflammatory proteins, protein breakdown/degradation, and lipid transport/removal^28,29^.

Interestingly, MYC and aryl hydrocarbon receptor nuclear translocator (ARNT) were predicted to be activated TFs in ACPs. The protooncogene MYC regulates the expression of numerous genes that control cell growth and cell cycle progression^30^. Moreover, MYC is activated by mitogenic signals such as WNT, SHH, and EGF signals^31^ in accordance with the activated signals in ACPs. ARNT is a transcription factor, also designated hypoxia-inducible factor (HIF)-1β, that plays a key role in the adaptive response to microenvironmental conditions such as toxic exposure and hypoxia^32^. Recent studies have shown that altered Wnt signaling in ACP may induce a tumor-specific cellular environment at the brain invasion border, which is consistent with the frequent finding of CNS invasion in ACP compared to PCP^33,34^. Furthermore, the outer tumor cell layer in ACP showed distinct expression of the neuroepithelial marker MAP2 compared to PCP^35^, which corresponds to our MS results. The development of these microenvironments is thought to be the response of the brain parenchyma to hypoxia^36^. Therefore, our results suggest that the hypoxic microenvironment in ACP might influence the activation of ARNT and the promotion of tumor cell migration^37,38^.

PCPs frequently harbor somatic *BRAF V600E* mutations^39^. BRAF plays a pivotal role in the MAPK pathway by promoting cell division, proliferation, and survival^40^. The V600E mutation enhances BRAF activity and leads to constitutive ERK activation^41^. Recently, BRAF V600E was found to influence the composition of the tumor microenvironment, modulating both immune cell infiltration and soluble mediators in thyroid and melanoma cancer^42–44^. In our network analysis, PCPs were associated with fatty acid metabolism, mitochondrial organization, exocytosis, cell adhesion, and inflammation, which are known to be associated with the immunosuppressive tumor microenvironment.

Cellular processes, including fatty acid metabolism and mitochondrial organization, control the immunosuppressive phenotype of tumor-associated macrophages, promoting tumor growth and metastasis by suppressing tumor immune surveillance^45^. Carnitine palmitoyltransferase 2 (CPT2), which is involved in mitochondria-dependent β-oxidation of long-chain fatty acids^46^, was significantly upregulated in PCP. In particular, the S100 protein family, including S100A2, S100A8, S100A9, S100A11, and S100A13, is upregulated in PCP and plays important roles in inflammatory responses by modulating the migration and infiltration of immunosuppressive cells, such as macrophages and neutrophils^47^. Additionally, RAB27B is involved in neutrophil migration and primary granule exocytosis^48^. Furthermore, our results were consistent with the histological characterization of PCP with scattered immune cells, including macrophages and neutrophils^7^.

In the TF prediction analysis, ELK1 and ESSRA (ERR1) were identified as activated TFs. The BRAF V600E mutation in PCP may phosphorylate MAPKs to activate ELK1 and ESSRA^49,50^. Interestingly, ELK1 plays a role in the regulation of immune cells in tumor microenvironments^51,52^. Moreover, ERR1 is associated with the regulation of the balance between tumor cytolytic lymphocytes and immunosuppressive M2 macrophages in melanomas^53^. These results support the supposition that BRAF V600E signaling is sufficient to recruit immunosuppressive cells into the tumor microenvironment, establishing a role for BRAF V600E in PCP as a tumor-intrinsic mediator of tumor immune escape and tumorigenesis.

Our results present, for the first time, molecular evidence at the protein level for the distinct genetic differences in CP subtypes. Despite those strengths of our study, our research also has some inherent limitations. First, our sample cohort consisted of unbalanced data as well as small sample size. In rare diseases, the number of patients might be extremely limited. Although the sample for the proteomic analysis was relatively small and unbalanced, our goal was to identify highly discriminatory proteomic features that function in a well-defined sample cohort. In addition, we simply performed validation experiments using small number of proteins to verify MS-based quantification. Although, this validation strategy is fairly common approach in the proteomic field, it could not confirm the biologically relevant finding in this study. Therefore, additional validation for proteins with important biological information at larger sample sizes will be important to confirm our proteomics findings.

Nevertheless, our quantitative proteomic analysis may provide insights into the development of diagnostic and prognostic biomarkers to support clinical decision-making. Because a better understanding of the molecular pathology of CP is of major importance for the development of targeted therapies aiming to improve the outcomes of patients with CP, our findings can further drive efforts toward emerging therapies that are appropriate for the molecular subtypes of CP.

## Materials and methods

### Patient samples

Institutional review board of Seoul National University Hospital approved this study (No. 1503-040-654 and 2011-135-1174) and all tumor samples were collected from the patients with the informed consents. All methods were carried out following relevant guidelines and regulations.

The tumor samples were obtained from the patients who gave informed consent and were deposited in a liquid nitrogen freezer (− 70 ℃) immediately after tumor removal. We matched the adamantinomatous tumor samples only by age and sex due to the rarity of papillary types.

We collected 9 ACP and 7 PCP samples from the tissue bank and prepared the samples. In preparation, 3 adamantinomatous and one papillary tumor sample were discarded due to poor sample quality or small sample size. In stage of sample preparation for proteomic analysis, 6 adamantinomatous and 6 papillary CPs were included.

### Tissue preparation

Frozen tissue samples were washed three times with PBS to remove blood contamination. Each tissue sample was lysed by homogenization with lysis buffer (4% SDS; 2 mM TCEP; and 0.1 M Tris–HCl, pH 7.4) and heated at 95 °C for 30 min. The protein concentration was determined using a reducing agent-compatible BCA assay kit (Thermo Fisher Scientific, Waltham, MA, USA). To eliminate contaminants from the samples, 250 µg of protein was precipitated with a fivefold volume of cold acetone. After centrifugation, the supernatant was removed, and the pellet was dried. Protein digestion was performed with trypsin according to a filter-aided sample preparation (FASP) protocol as described previously^54,55^. The protein pellets were resuspended in 50 µl of reduction buffer (4% SDS; 0.1 mM DTT; and 0.1 M Tris–HCl, pH 8.5) and heated at 95 °C for 15 min. Reduced samples were mixed with 300 µl of UA solution (8 M urea in 0.1 M Tris–CL, pH 8.5). The protein samples were loaded onto a Microcon Ultracel 30 kDa filter (Millipore, Billerica, MA, USA) and centrifuged at 14,000×*g* at 21 °C for 15 min. Buffer was exchanged twice with 400 µl of UA solution. Then, the reduced cysteines were alkylated with 0.05 M iodoacetamide in UA buffer for 30 min at room temperature in darkness. The filter was washed twice with 400 µl of UA buffer, followed by a triple buffer exchange with 400 µl of 50 mM tetraethylammonium bicarbonate (TEAB). Finally, trypsin (Promega, Madison, WI) in 50 mM TEAB was added to each sample. The protein to enzyme ratio was 100:1. The samples were incubated overnight at 37 °C, and the peptides were eluted by sequential centrifugation. The peptide concentrations were quantified by tryptophan fluorescence assay^56^.

### C18 StageTip peptide desalting

For label-free quantitation, 20 µg of peptides were acidified with 10% trifluoroacetic acid (TFA), and the acidified peptides were loaded onto an in-house-prepared Stage-Tip with SDB-RPS disk in a 200-µl pipet tip. For column washing, we used 100 µl of 0.2% TFA three times. The peptides were eluted with elution buffers (elution buffers 1, 2, and 3) into three fractions. All the eluted peptides were dried in a speed vacuum.

### TMT labeling

Tandem mass tag (TMT) labeling was performed according to the manufacturer’s protocol with some modifications. Briefly, TMT 10-plex (Thermo Fisher Scientific, Waltham, MA, USA) reagent (0.8 mg) was dissolved in 100% ACN. Each 40-µg sample was spiked with 260 ng of peptides derived from ovalbumin for use as an internal standard, and ACN was added to the reagent to give a final concentration of 30% (v/v). After incubation at room temperature for 1 h, the reaction was quenched with 5% hydroxylamine. The TMT-labeled peptides were pooled at a 1:1:1:1:1:1:1:1:1:1 ratio, and the mixtures were dried in a speed vacuum.

### Offline high-pH reversed-peptide fractionation

The labeled tryptic peptides were fractionated offline using the reversed-phase high-pH strategy as described previously^57^. Before high-pH fractionation, the pooled peptides were desalted using Oasis solid phase extraction (SPE) columns (Waters, Milford, MA, USA), and the resulting peptides were subjected to Agilent 1290 bioinert HPLC (Agilent, Santa Clara, CA, USA) equipped with an analytical column (4.6 × 250 mm, 5 μm). For peptide separation, buffer A consisted of 15 mM ammonium hydroxide, and buffer B consisted of 15 mM ammonium hydroxide in 90% ACN. The peptides were fractionated with a gradient from 5 to 35% ACN at a flow rate of 0.2 ml/min. A total of 96 fractions were concatenated into 24 fractions and evaporated in a speed vacuum.

### LC–MS/MS analysis

All samples were analyzed by LC–MS/MS using quadrupole orbitrap mass spectrometers, Q-Exactive Plus (Thermo Fisher Scientific, Waltham, MA, USA) coupled with an Ultimate 3000 RSLC system (Dionex, Sunnyvale, CA, USA) consisting of EASY-Spray™ LC columns with an electrospray source, and the temperature of the column heater was set to 60 °C^57^. Peptides were separated on a two-column system with a trap column (5 mm in length and 300 µm in diameter) and an analytic column (EASY-Spray C18, 75 µm I.D. × 50 cm length, 2 µm). A gradient was applied using 0.1% formic acid in water as solvent A and 0.1% formic acid in ACN as solvent B. The mass spectrometer was operated in data-dependent acquisition mode. The survey MS scan (350 to 1650 m/z) was acquired at a mass resolution of 70,000 at m/z 200, and the MS/MS spectrum was acquired at a mass resolution of 17,500 at m/z 200. For label-free analysis, the tandem mass spectra of the 15 most abundant peaks were acquired by peptide fragmentation using high collision dissociation (HCD). The normalized collision energy (NCE) was set to 28% with an isolation window of 1.2 m/z. In the cases in which quantification was based on TMT, the 20 most abundant peptide ions in the full MS scan were also fragmented using a higher HCD (NCE 32% with isolation width of 1.2 m/z).

### Data processing

In the case of label-free quantification, MaxQuant (ver. 1.6.1.0) software^58^ was used to perform a database search. MS Raw files were searched against the Humans UNIPROT protein sequence database (released December 2014 with 88,657 entries) using the Andromeda search engine^59^. Mass tolerances were set to 6 ppm and 20 ppm for the precursor and fragment ions, respectively. Cysteine carbamidomethylation and methionine oxidation were considered fixed and variable modifications, respectively. Full tryptic digestion was chosen with two missed cleavages allowed, and peptides with at least six amino acids were considered for identification. The false discovery rate (FDR) was set to 1% for the peptide, protein and modification levels. To increase the number of features for quantifying samples, we selected the ‘Match between runs’ option in MaxQuant.

For the database search for the TMT 10-plex experiment, raw MS files were processed using Proteome Discoverer ver2.2 with the SEQUEST-HT algorithm against the Human UniProt protein sequence database (December 2014, 88,657 entries). The search parameters included full enzyme digestion using trypsin with up to two missed cleavages, a 20-ppm peptide precursor mass tolerance and a fragment ion mass tolerance of 0.02 Da. Variable modifications of 15.995 Da for methionine oxidation and 42.011 Da for protein N-term acetylation and fixed modifications of 57.021 Da for carbamidomethylation on cysteine residues and 229.153 Da for TMT 10-plex-labeled lysine, and any N-terminus was selected. The coisolation threshold for quantification of the peptides was set to 50%. The FDRs of peptide-spectral matches (PSMs) and proteins were set to be less than 1%.

MS-based proteomics data of all identified peptides and proteins listed were deposited in the ProteomeXchange Consortium (http://proteomecentral.proteomexchange.org) via the PRIDE partner repository: dataset identifier PXD023376 (for Label-free quantification) and PXD028790 (for TMT quantification).

### Statistical analysis

Perseus software was used for all statistical analyses^15^. In the case of label-free analysis, the intensity-based absolute quantification (iBAQ) algorithm was used as part of the MaxQuant platform^60^. To ensue that normally distributed data were obtained, the iBAQ intensities were log2-transformed. After filtering based on “reverse” and “only identified by site” and ensuring that at least 70% of the values were valid in the two groups, missing values were replaced separately in each sample based on a normal distribution using a width of 0.3 and downshift of 1.8. Using a width adjustment, the data were normalized. Proteins with a permutation-based FDR < 0.1 (two-sample Student’s t-test) and fold change > 1.5 were considered significantly expressed.

The analysis for TMT-based quantification was based on the logarithmic (log2(x)) intensities of TMT-reporter ions. After filtering out “reverse” and “only identified by site” candidates, the data were normalized as in the label-free analysis. Proteins with a permutation-based FDR < 0.1 (two-sample Student’s t-test) and fold change > 1.2 were considered to be significantly expressed.

### Bioinformatics analysis

Gene ontology (GO) enrichment analysis in the biological process (BP) category was performed using the DAVID bioinformatics tool^61^. For visualization of the predicted associations for significantly expressed proteins, the STRING database (ver. 11.0) was used^62^. For visualization of the heat map for hierarchial clustering, Perseus software was used. Transcription factor binding site (TFBS) prediction was performed using Toppgene (https://toppgene.cchmc.org/enrichment.jsp)^63^.

### Western blotting

Proteins were extracted from tissues using lysis buffer (4% SDS; 2 mM TCEP; and 0.1 M Tris–HCl, pH 7.4). Insoluble pellets were removed by centrifugation at 15,000 rpm at 21 °C for 20 min. Lysates containing 10 µg of protein were loaded on SDS-PAGE gel and subsequently blotted onto a PVDF membrane (Millipore, Darmstadt, Germany). After blocking for 2 h with 5% bovine serum albumin (BSA) in TBS-T buffer, the membrane was incubated overnight at 4 °C with one of the following antibodies: anti-beta-actin (1:2000 dilution; sc-47778; Santa Cruz, Dallas, TX, USA), anti-EPCAM (1:1000 dilution; sc-21792; Santa Cruz, Dallas, TX, USA) and anti-P4HB (1:1000 dilution; 2446; Cell Signaling, Danvers, MA, USA). After three washing steps with TBS-T buffer, the membrane was incubated for 2 h with a secondary anti-mouse (1:5000 dilution; 31430; Invitrogen, Carlsbad, CA, USA) or anti-rabbit (1:5000 dilution; 31460; Invitrogen, Carlsbad, CA, USA) antibody and washed again three times with TBS-T buffer. Finally, protein expression on the membranes was measured by a Amersham Imager 680 blot and gel imager (GE, Boston, MA, USA) using a SuperSignal™ West Femto maximum sensitivity substrate (Thermo Fisher Scientific, Waltham, MA, USA).

### Ethics declaration

This study was approved by the institutional review board (No. 1503-040-654 and 2011-135-1174) and all tumor samples were collected from the patients with the informed consents.

## Supplementary Information

Supplementary Figure S1.

Supplementary Figure S2.

Supplementary Figure S3.

Supplementary Figure S4.

Supplementary Figure S5.

Supplementary Table S1.

Supplementary Table S2.

Supplementary Table S3.

Supplementary Table S4.

Supplementary Table S5.

Supplementary Table S6.

Supplementary Table S7.

Supplementary Table S8.

Supplementary Table S9.

Supplementary Information 15.

Supplementary Information 16.

Supplementary Information 17.

## Data Availability

Some or all data generated or analyzed during this study are included in this published article or in the data repositories listed in References.
