# Supplementary material for: In-depth proteomic profiling captures subtype-specific features of craniopharyngiomas
Source: Sci Rep. 2021 Oct 27;11:21206. doi: 10.1038/s41598-021-00483-4 (PMC8551227; doi:10.1038/s41598-021-00483-4)
Supplement: Supplementary file 2 — Supplementary Figure S2. [file 41598_2021_483_MOESM2_ESM.pptx]

## Slide 1
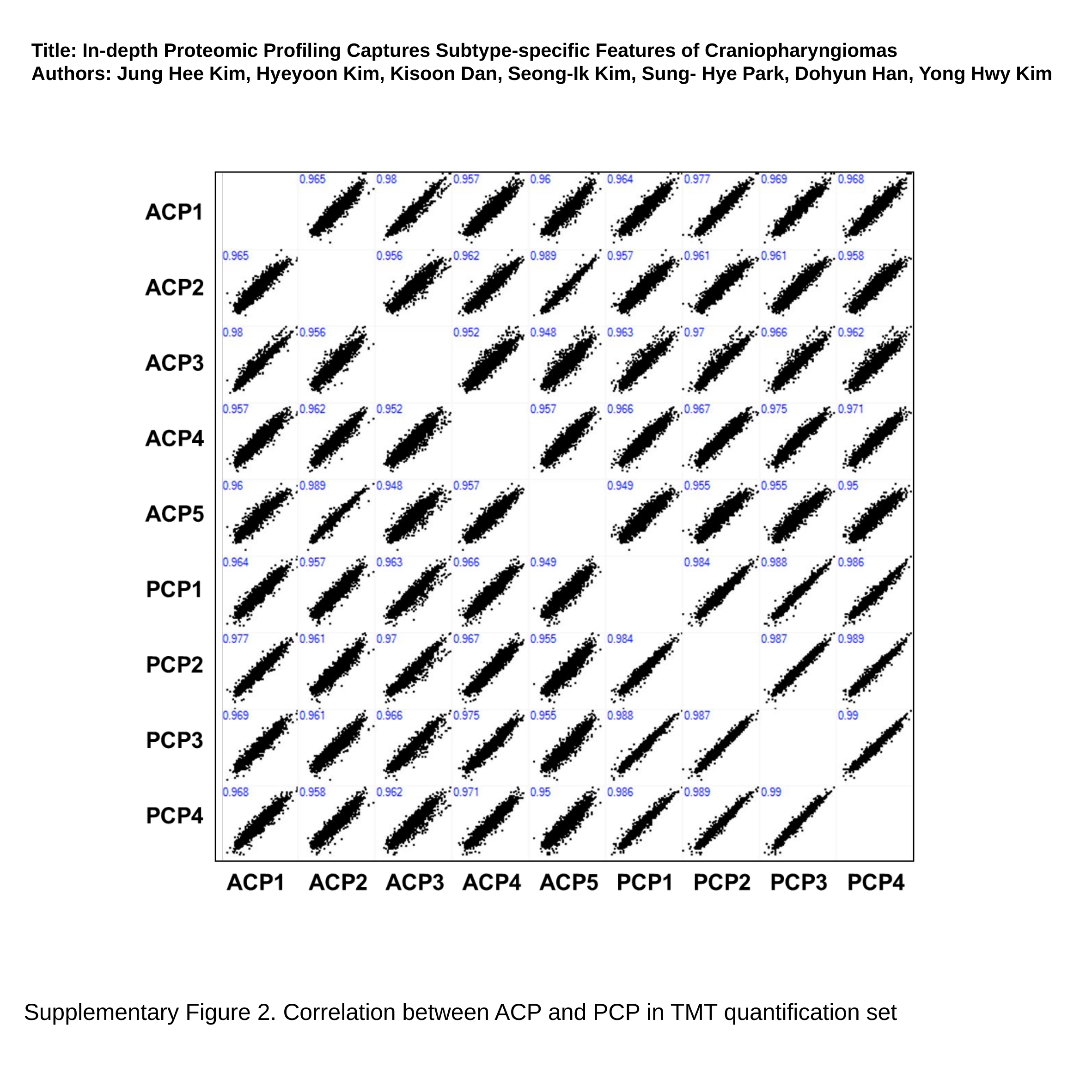

Title: In-depth Proteomic Profiling Captures Subtype-specific Features of Craniopharyngiomas
Authors: Jung Hee Kim, Hyeyoon Kim, Kisoon Dan, Seong-Ik Kim, Sung- Hye Park, Dohyun Han, Yong Hwy Kim
Supplementary Figure 2. Correlation between ACP and PCP in TMT quantification set
