# Supplementary figures and images for: In-depth proteomic profiling captures subtype-specific features of craniopharyngiomas
Source: Sci Rep. 2021 Oct 27;11:21206. doi: 10.1038/s41598-021-00483-4 (PMC8551227; doi:10.1038/s41598-021-00483-4)

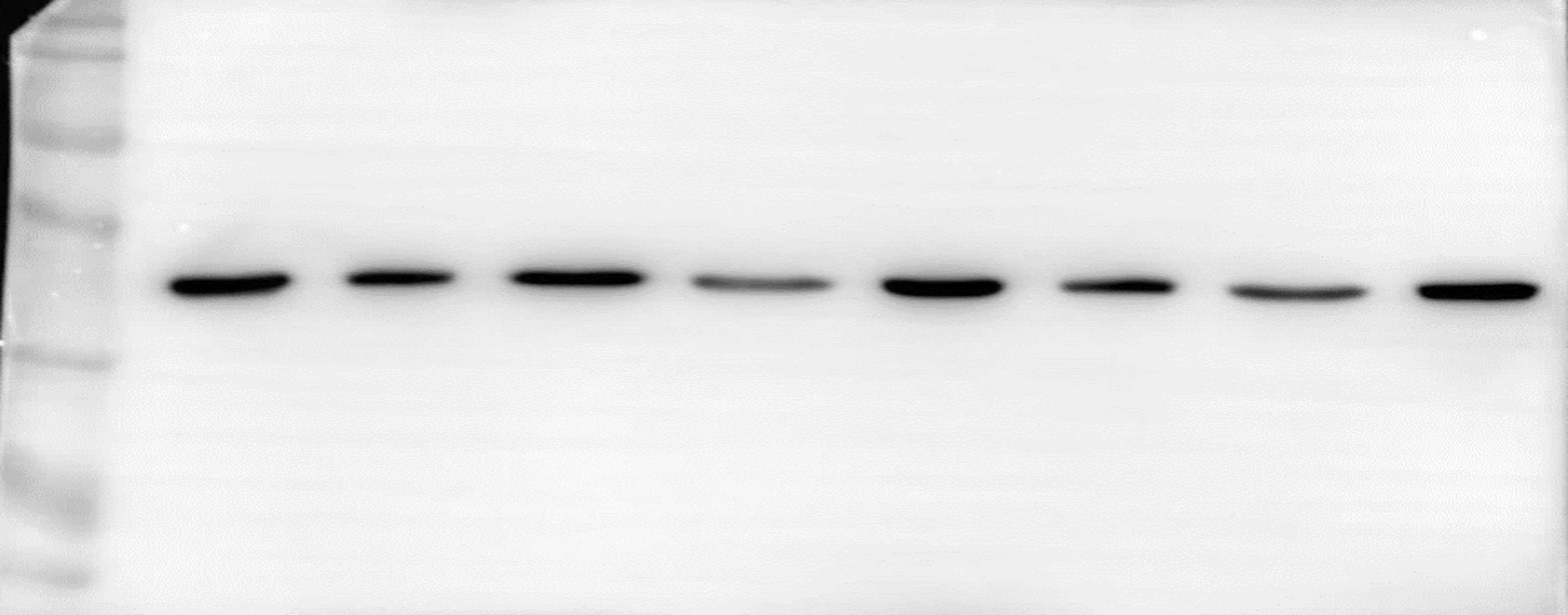

Supplement: Supplementary file 15 — Supplementary Information 15. [file 41598_2021_483_MOESM15_ESM.tif]

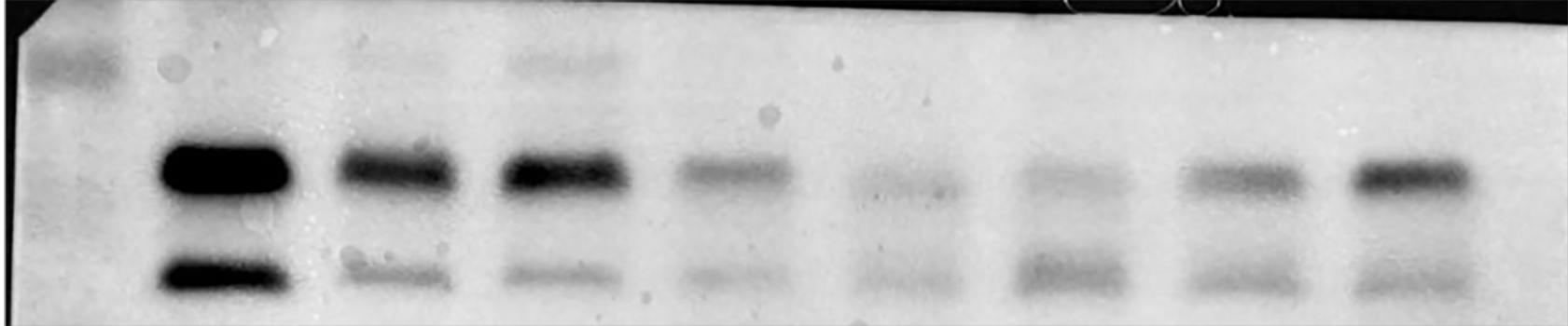

Supplement: Supplementary file 16 — Supplementary Information 16. [file 41598_2021_483_MOESM16_ESM.tif]

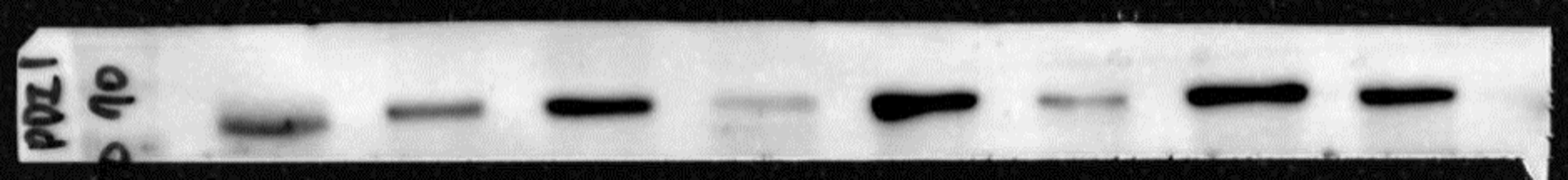

Supplement: Supplementary file 17 — Supplementary Information 17. [file 41598_2021_483_MOESM17_ESM.tif]
